# Stereotactic radiosurgery (SRS) in the modern management of patients with brain metastases

**DOI:** 10.18632/oncotarget.7131

**Published:** 2016-02-02

**Authors:** Hany Soliman, Sunit Das, David A. Larson, Arjun Sahgal

**Affiliations:** ^1^ Department of Radiation Oncology, Odette Cancer Centre, Sunnybrook Health Sciences Centre, University of Toronto, Toronto, ON, Canada; ^2^ Division of Neurosurgery, St. Michaels Hospital, University of Toronto, Toronto, ON, Canada; ^3^ Department of Radiation Oncology, University of California San Francisco, San Francisco, CA, USA

**Keywords:** stereotactic radiosurgery, brain metastases, whole brain radiation, targeted therapy

## Abstract

Stereotactic radiosurgery (SRS) is an established non-invasive ablative therapy for brain metastases. Early clinical trials with SRS proved that tumor control rates are superior to whole brain radiotherapy (WBRT) alone. As a result, WBRT plus SRS was widely adopted for patients with a limited number of brain metastases (“limited number” customarily means 1-4). Subsequent trials focused on answering whether WBRT upfront was necessary at all. Based on current randomized controlled trials (RCTs) and meta-analyses comparing SRS alone to SRS plus WBRT, adjuvant WBRT results in better intracranial control; however, at the expense of neurocognitive functioning and quality of life. These adverse effects of WBRT may also negatively impact on survival in younger patients. Based on the results of these studies, treatment has shifted to SRS alone in patients with a limited number of metastases. Additionally, RCTs are evaluating the role of SRS alone in patients with >4 brain metastases. New developments in SRS include fractionated SRS for large tumors and the integration of SRS with targeted systemic therapies that cross the blood brain barrier and/or stimulate an immune response. We present in this review the current high level evidence and rationale supporting SRS as the standard of care for patients with limited brain metastases, and emerging applications of SRS.

## INTRODUCTION

Brain metastases are a significant cause of morbidity and mortality in patients with metastatic cancer, with an incidence of up to 65% during the course of illness [1, 2]. The most common primary sites are lung, melanoma, renal, breast and colorectal cancer [3]. Options for patients with brain metastases had been limited to whole brain radiotherapy (WBRT) or supportive care alone, and systemic chemotherapy was often discontinued. The development of brain metastases was viewed as an oncologic terminal event.

As systemic therapies have become more efficacious in patients with metastatic disease, improved survival rates are now being observed. In addition, the patterns of disease progression are shifting such that the incidence of brain metastases is increasing while extra-cranial disease remains controlled. This phenomena is likely a consequence of the central nervous system (CNS) being a sanctuary site from drug penetration. As a result, the management of brain metastases has become a major focus of research, with the intent to improve intra-cerebral control and decrease neurologic deaths.

Although the role of neurosurgery had been established in the 1990s as a means to achieve local control and prolong survival, it was reserved for the minority of patients presenting with a single metastasis and no other disease beyond the brain [4-7]. Still lacking was a focal ablative non-invasive treatment that could be applied efficiently to a much broader population of patients with brain metastases. This set the stage for the development of stereotactic radiosurgery (SRS). SRS is a focused ablative radiation treatment delivered with sub-millimeter precision to the tumor localized in three-dimensions in 1-5 fractions.

The focus of this review is to summarize the current high level evidence to clarify the role of SRS as optimal management for patients presenting with limited brain metastases. Furthermore, we provide an overview of the emerging applications of SRS as it continues to evolve into a treatment alternative to WBRT, with the intent to maximize neurcognitive function and quality of life (QOL).

## PATHOPHYSIOLOGY OF BRAIN METASTASES

Circulating tumor cells (CTCs) can disseminate from a primary tumor mass to form distant colonies through implantation at an ectopic site, such as the brain [8-10]. To do so requires CTCs to arrest within the circulation, extravasate from the bloodstream or lymphatics into the brain, and survive and proliferate [11-13]. The process of metastatic colonization involves a direct interaction of CTCs with endothelial cells and astrocytes in the brain microenvironment. For example, CTCs that have arrested within the capillary bed direct local endothelial cells to remodel the adjacent environment to promote tumour cell growth and invasion [14-16]. Further, metastatic tumour cells recruit systemic stromal cells, such as fibroblasts, to assist with early colonization [17], and co-opt local stromal cells, such as reactive astrocytes and microglia, to promote tissue invasion [15, 18, 19]. Astrocytes within the tumour microenvironment may also play a role in protecting tumour cells from chemotherapy-induced cytotoxicity, through a yet to be defined mechanism requiring cell-cell contact [20, 21]. Tumour cells, through release of migration inhibitory factor, interleukin-8, and plasminogen activator inhibitor 1, induce astrocyte activation and modify the inflammatory milieu to enhance tumor-cell proliferation *in vitro* [22-24]. These molecular processes represent novel and understudied possible therapeutic targets for the treatment of intracranial metastatic disease.

## RADIOBIOLOGY OF SRS

A typical SRS dose of 20Gy delivered in 1 treatment is substantially more than the biologically equivalent dose (BED) of a commonly prescribed WBRT dose of 30Gy in 10 fractions. However, the greater BED alone may not explain the superior control and response rates inherent to SRS. It is postulated that additional biologic factors or cellular pathways specific to high dose per fraction radiation may be involved in the pathophysiology of SRS response. In particular, activation of the acid sphingomyelinase pathway has been shown to occur only when the dose per fraction increases beyond 8 Gy, and serves to activate tumor endothelial cell apoptosis, disrupt the tumor vasculature and increase tumor cell death[25]. In addition, release of tumor-specific antigens leading to the priming of CD8+ T cells and a subsequent immune mediated response may further enhance tumor cell death again specific to SRS dosing [26]. The radiobiology specific to SRS is an area of active research [27].

## PROGNOSTIC SCORING SYSTEMS

SRS was initially a very resource intensive therapy offered only at specialized centers and indicated only for metastatic patients with a good life expectancy. The challenge lay in prognosticating patients effectively and as a result the Radiation Therapy Oncology Group (RTOG) recursive partitioning analysis (RPA) [28, 29] was developed. Based on the patient's Karnofsky Performance Status (KPS), age, status of the primary tumor and presence of extracranial disease, patients were grouped into class 1, 2 or 3 with corresponding median survivals of 7.1, 4.2 and 2.3 months, respectively. Although a major development at the time, the RPA is now considered overly simplistic as current oncologic decision making is far more complex incorporating molecular, histological, clinical and radiographic disease characteristics. There are now more sophisticated classification tools, such as the diagnosis-specific graded prognostic assessment (DS-GPA). This system provides histology-specific estimates of survival and can separate, for example, the most favorable breast cancer patients with an expected survival of 25 months (excellent KPS and luminal B type breast cancer) from the least favorable patients with an expected survival of 3 months (poor KPS and basal-like breast cancer) [30]. Despite advances in prognostication of patients with brain metastases, physicians are still largely unable to accurately predict long-term survivors. A study asking expert physicians to estimate survival of a 150 patients with information about cancer type, number of brain metastases, neurological presentation, extra-cranial disease status, KPS, RPA class, prior whole-brain radiotherapy, and synchronous or metachronous presentation, showed that more than 45% of predictions were off by more than 6 months and 18% were off by more than 12 months [31]. Further advances in prognostic tests such as the “liquid biopsy” (a non-invasive blood test that can detect tumor DNA or RNA fragments or CTCs) are needed and in development [32]. These combine advanced patient and tumor specific genomic information into the equation, in order to achieve personalized survival predictions.

## THE NON-SURGICAL MANAGEMENT OF 1-4 BRAIN METASTASES - LEVEL 1 EVIDENCE

Surgery continues to be an important treatment option for patients with limited brain metastases. It is indicated when metastases are large (>3-4 cm), or when a pathologic diagnosis is needed. In addition, surgery is preferred in the presence of significant edema requiring prolonged high dose dexamethasone, or to potentially reverse neurological deficits. Otherwise, the current evidence suggests that the efficacy of SRS is sufficient to achieve durable local control that is comparable to surgery. Although there are no RCTs directly comparing the two, several trials have been reported comparing SRS to SRS with WBRT (Table 1), and is a major focus of this review.

**Table 1:** Summary of the randomized trials involving SRS and WBRT

RCT	Patients included	% Single brain tumors	Primary Endpoint	Local control	Distant control	Overall Survival	Functional Outcomes	Radiation Necrosis
Kondziolka et al.^34^ WBRT + SRS (N=13) vs. WBRT (N=14)	2-4 brain metastases, diameter<2.5cm	NA	Local Control	92% vs 0% at 1 yr (p=0.0016)	NR	Median: 11mos vs 7.5mos (NS)	NR	NR
Andrews et al.^34^ RTOG 9508 WBRT + SRS (N=164) vs. WBRT (N=167)	1-3 brain metastases, KPS≥70, maximum diameter 4cm	56 % vs. 56 %	Overall Survival	82% vs.71% at 1 yr (p=0.01)	NR	SingleMets: Median 6.5mos vs. 4.9mos (p=0.04) Multiple mets (NS)	No difference in mental status More patients KPS improved with WBRT and SRS (12%) vs WBRT alone (4%) p=0.03	NR
Aoyama et al.^35^ JRSOG99-1, SRS (N=67) vs. WBRT+SRS (N=65)	1-4 metastases, KPS≥70, maximum diameter 3 cm	49% vs. 48%	Brain tumor recurrence	72.5% vs. 88.7% at 1 yr (p=0.002)	36.3% vs. 58.5% at 1 yr (p=0.003)	28.4% vs. 38.5% at 1 yr (p=0.42)	No difference in MMSE or neurologic functional preservation	Grade 4: SRS alone 1 case and SRS and WBRT 2 cases
Chang et al.^36^ SRS (N=30) vs. WBRT +SRS (N=28)	1-3 metastases, RPA 1 or 2 (KPS≥70)	60% vs. 54%	Neuro-cognition: HVLT-R total recall at 4 mos	67% vs. 100% at 1 yr (p=0.012)	45% vs. 73% at 1 yr (p=0.02)	63% vs. 21% at 1 yr (p=0.003)	HVLT-R total recall mean posterior probability of decline: SRS and WBRT 52% SRS alone 24%	2 cases of grade 4 in SRS alone arm
Kocher et al.^37^ EORTC 22952-26001, SRS (N=100) vs. WBRT+SRS (N=99)	1-3 metastases WHO performance status ≤2, stable disease or asymptomatic synchronous primary tumor	68% vs. 66%	Duration of functional independence based on a WHO >2	69% vs. 81% at 2 yr (p=0.04)	52% vs. 67% at 2 yr (p=0.023)	Median OS (including surgical patients): 10.9mos vs. 10.7mos (p=0.89)	*No difference in time to WHO>2 in patients who had WBRT (10.0 mos) vs observation (9.5 mos)	SRS alone: 8% SRS and WBRT: 13%
Brown et al.^38^ NCCTG N0574 (Alliance) SRS VS WBRT + SRS (N=213)	1-3 metastases, diameter<3cm	55% vs. 50%	Decline >1SD from baseline in any of 6 cognitive tests at 3 months	Intracranial control at 6 and 12 months - SRS: 66.1% and 50.5% WBRT + SRS: 88.3% and 84.9%		Median OS: 10.7 mos vs 7.5 mos	Decline >1SD at 3 months more frequent in WBRT + SRS (31% vs 8%) p=0.007	NR

HR: hazard ratio, WHO: world health organization, KPS: Karnofsky performance status, WBRT: whole brain radiotherapy, SRS: stereotactic radiosurgery, yr: year, mos: months, NS: not significant, NR: not recorded, NA: not applicable, HVLT-R: Hopkins Verbal Learning Test revised.

* Patients in the observation group had either surgery alone or SRS alone. Functional outcome was not analyzed individually by surgery or SRS alone.

The first RCTs evaluating SRS mimicked the design of the initial surgical studies for brain metastases, and evaluated the addition of SRS to WBRT [33, 34]. These studies confirmed that SRS improved local control, and a survival advantage in selected patients with a single brain metastasis was shown. These trials were successful in shifting the paradigm from WBRT alone to WBRT plus SRS for patients presenting with limited brain metastases and a good performance status.

The next series of clinical trials were intended to answer if WBRT was at all necessary, and compared SRS alone to SRS with adjuvant WBRT. Three RCTs have been reported in patients presenting with up to 4 metastases [35-37] and a forth in abstract form [38]. Aoyama et al. [35] reported the first RCT, randomizing 132 patients to SRS alone (65 patients) or WBRT plus SRS (67 patients). The primary endpoint was brain tumor recurrence. Although the 1-year local control rate was high with SRS alone at 73%, additional local control with adjuvant WBRT at 89%, was observed. Furthermore, adjuvant WBRT reduced the rate of distant intra-cranial relapse at 1 year from 64% to 42%. However, these gains in intra-cranial control did not translate into an advantage with respect to cognition, based on Mini-Mental Status Exam (MMSE), nor survival. Moreover, patients had higher rates of necrosis and leukoencephalopathy in the WBRT arm. The trial was thus successful in providing the first level 1 evidence to clarify the relative impact of SRS alone versus SRS with WBRT.

Rather than the traditional endpoint of survival or intra-cranial control, Chang et al. [36] took a different approach and evaluated neurocognition as the primary endpoint. Importantly, the validated Hopkins Verbal Learning Test-Revised (HVLT-R) assessment tool, was used to measure neurocognitive functioning as opposed to the MMSE which is not a sensitive test for neurocognition [39]. Fifty-eight patients were randomized to SRS alone (*n* = 30) or WBRT plus SRS (*n* = 28). Early stopping rules were invoked at the interim analysis, and the study concluded that SRS alone was favored with respect to the probability of neurocognitive decline at 4 months post-treatment. This benefit was realized despite the 1 year local control and distant brain control rates favoring adjuvant WBRT with absolute gains of 33% and 27%, respectively. The intracranial control outcomes were in keeping with the results from the Aoyama study, but what was not expected was the survival advantage observed in the SRS alone arm. The median survival was 15 months in the SRS alone arm and 6 months in the SRS with WBRT arm (*p* = 0.003). This survival outcome caused the trial to undergo considerable scrutiny with many arguing that the survival advantage for SRS alone was a result of imbalances between the two arms, as more patients treated with SRS plus WBRT had greater extra-cranial (more liver and adrenal metastases) and intracranial disease (larger volume of brain metastases) [40].

The European Organization for Research and Treatment of Cancer (EORTC) also took a different approach with their primary endpoint focusing on functional independence [37]. They utilized, however, the more clinically familiar World Health Organization (WHO) performance status (PS) scale, and measured the time to WHO PS deterioration to more than 2. This RCT concluded that the addition of WBRT did not improve the median duration of functional independence (SRS alone: 10.0 months vs SRS plus WBRT: 9.5 months). The secondary outcome of QOL, measured with the validated EORTC-QLQC30 tool, was observed to be worse in patients who received WBRT in several QOL domains [41]. Importantly, these results were observed despite adjuvant WBRT reducing the 2-year local failure rate (31% to 19%), distant brain failure (48% to 33%), and the need for salvage therapies (51% *vs*. 16%). No significant differences in survival were observed albeit the trial was not powered to address survival.

The most recent trial, NCCTG N0574 [38], presented by Brown et al. in the 2015 American Society of Clinical Oncology annual meeting has put an end to the debate of whether WBRT should be added to SRS in patients with a limited number of brain metastases. Two-hundred and thirteen patients with 1 to 3 brain metastases up to 3 cm in size were randomized to SRS or SRS plus WBRT. The primary endpoint was cognitive progression from baseline in any of the 6 cognitive tests conducted at 3 months. Cognitive progression at 3 months was more frequent after SRS plus WBRT vs SRS alone (88.0% vs 61.9% respectively, *p* = 0.002). There was more deterioration in the SRS+WBRT arm in immediate recall (31% vs 8%, *p*= 0.007), delayed recall (51% *vs* 20%, *p* = 0.002), and verbal fluency (19% *vs* 2%, *p* = 0.02). Intracranial tumour control at 6 and 12 months favoured SRS plus WBRT (*p* < 0.001). Median overall survival (OS) were statistically nonsignificant at 10.7 months for SRS alone vs 7.5 months for SRS+WBRT respectively (HR = 1.02, *p* = 0.93).

These four trials have established the role of SRS alone as the standard of care in patients with a limited number of brain metastases, but they have not been designed to evaluate the impact of WBRT on overall survival.

## META-ANALYSES

The inclusion criteria across the RCTs evaluating SRS alone to SRS plus WBRT were similar (Table 1), however, the primary endpoints were completely different, and no trial was designed to evaluate overall survival. To better clarify the outcomes of local control, distant brain control and survival, a meta-analysis (MAL) was performed by Tsao, Xu and Sahgal in 2012 [42]. They reported hazard ratios for local control and distant brain control favoring adjuvant WBRT at 2.61 and 2.15, respectively, but were unable to combine the survival results due to limitations in the way the data were reported. Sahgal et al. then performed an individual patient data (IPD) MAL of the same RCTs aimed at evaluating treatment effects on survival, and perform sub-group analyses [43]. With respect to overall survival, a treatment effect was observed favoring SRS alone in patients ≤50 years. In older patients, no survival disadvantage was observed with SRS alone. With respect to distant brain control, a treatment effect was also observed with risk reductions in the development of new brain metastases in older patients treated with WBRT, but no benefit in the younger patients (≤50 years) treated with WBRT. As expected, local control was improved with additional WBRT in all age groups. The concordance between a survival detriment and lack of benefit in distant brain control despite treatment with WBRT, in the younger patients, led the authors to hypothesize that exposure to the known harms of WBRT (discussed in detail below) may negatively impact patient survival. This hypothesis is provocative, and remains to be validated.

## TOXICITY OF WBRT

The most convincing trial design to confirm the adverse effects of WBRT is to randomize patients with no visualized brain metastases to observation or WBRT. This has been done in studies evaluating prophylactic cranial irradiation (PCI) in both small cell lung cancer (SCLC) [44, 45] and non-small cell lung cancer (NSCLC) [46, 47]. PCI, not dissimilar to the addition of WBRT to SRS, has been shown to reduce the risk of intra-cranial relapse. However, this gain comes at the expense of a negative effect on QOL. For example, significant increases in fatigue, appetite loss, nausea and vomiting and leg weakness were observed in patients with extensive stage SCLC at 6 and 12 weeks post PCI [48]. Furthermore, in locally advanced NSCLC patients, a greater decline in HVLT-R measures of immediate and delayed recall were observed even up to 1 year post-PCI [46]. With respect to longer-term adverse effects of WBRT, a devastating consequence can be the development of leukoencephalopathy. Indeed, radiation-induced dementia rates have been reported to be as high as 11% in long-term brain metastases survivors (>12 months) after WBRT [49], and reported to be greater in patients treated with WBRT and SRS as opposed to SRS alone [35, 50].

## MECHANISM OF HARM

Excessive N-methyl-D-aspartate (NMDA) receptor stimulation, similar to the pathophysiology inherent to Parkinson's dementia [51], has been postulated as one mechanism explaining the adverse neurocognitive effects of WBRT. As a strategy to mitigate the risk, RTOG 0614 [52] evaluated memantine, a neuroprotective agent that blocks pathologic stimulation of NMDA, in a large randomized placebo controlled trial involving 554 patients. At 24 weeks, 64% of patients without memantine and 54% with memantine had cognitive function failure based on assessment with the HVLT-R. Although the primary endpoint did not reach significance due to the high attrition rate, there was a strong trend (*p* = 0.059) supporting memantine treatment. Analysis of secondary endpoints showed that memantine significantly prolonged the time to cognitive decline and yielded superior results for executive function, processing speed and delayed recognition at 24 weeks. Ultimately, these data show the majority of patients suffer cognitive dysfunction with WBRT, and that pharmacologic strategies to mitigate the risk by targeting similar pathways involved in Parkinson's dementia may be worthy of further investigation.

Further insight into WBRTs’ mechanism of damage has come from a recent Phase 2 trial evaluating hippocampal avoidance WBRT (HA-WBRT). Sophisticated modern radiation technology was applied to limit dose exposure to the hippocampus to no more than 80% of the prescribed 30Gy in 10 fractions [53]. This non-randomized phase 2 trial measured neurocognitive function using the HVLT-R, and compared outcomes to historic controls treated with conventional WBRT from a RTOG trial database. HA-WBRT resulted in a 7% decline in the mean relative HVLT-R delayed recall, which was significantly lower than the 30% decline obtained from historic controls. Although this study is encouraging in terms of limiting toxicity from WBRT, a randomized trial is required to fully understand the therapeutic value in this technologically complex form of WBRT.

## WHERE DO WE STAND FOR THE PATIENT PRESENTING WITH LIMITED BRAIN METASTASES?

In 2012, the American Society for Therapeutic Radiation Oncology published an evidence-based guideline with a series of recommendations on the management of brain metastases [54]. Since that time, the evidence from clinical trials has made it clear that the preferred up-front radiation treatment option for patients with limited brain metastases is SRS alone. In fact, the American Society for Radiation Oncology (ASTRO) has recently made a definitive recommendation in their Choose Wisely campaign and recommend to not routinely add adjuvant WBRT to SRS for limited brain metastases [55]. Moreover, the recently revised National Comprehensive Cancer Network (NCCN) recommendations now support SRS alone even beyond those patients with “limited” metastases, and do not specify an upper limit for the number of metastases [56]. The latter is forward thinking; metastasis counting is likely irrelevant and what matters is otherwise the suitability of the patient for SRS [57-59]. Therefore, the onus is no longer on those who treat with SRS alone to justify the omission of WBRT; rather it is on those who treat with WBRT in a SRS suitable patient.

## WHAT ABOUT THE PATIENT WITH MORE EXTENSIVE BRAIN METASTASES?

Previous technical limitations in SRS technology restricted the application of SRS to patients with only a few brain metastases. Advancements in fixed frame and frameless SRS technologies now allow treatment of numerous metastases in a single session [60, 61].

The first major prospective study evaluating SRS alone for multiple brain metastases was reported in 2014 [62]. Approximately 1200 patients with 1-10 SRS eligible metastases were treated with SRS alone using Gamma Knife technology (Elekta AB, Stockholm, Sweden). The analysis was broken down according to the 455 patients with 1 metastases, 531 patients with 2 to 4 metastases and 208 with 5 to 10 metastases. Survival, distant brain relapse, and local control rates were not significantly different in patients with 5 to 10 metastases *versus* 2 to 4 metastases. These results are of major significance as they challenge the dogma that patients with more than 4 metastases will not benefit from SRS alone due to shortened survival and will inevitably fail elsewhere in the brain. Therefore, this trial provides evidence to support SRS alone in good KPS patients with up to 10 metastases, provided the individual tumor volume is no more than 10 mL and < 3 cm in longest diameter, and the total cumulative volume of all tumors in the brain is ≤15 mL. Several randomized trials are currently evaluating WBRT alone *versus* SRS alone in similar patients, and in development is a trial evaluating SRS alone *versus* WBRT plus SRS boost in patients with 10 to 20 metastases.

## TARGETED THERAPIES IN COMBINATION WITH BRAIN RADIATION

As compared to traditional chemotherapy, several targeted agents have higher penetration through the blood-brain barrier and yield response in the brain. Dramatic improvements in disease control are being observed even for histologies such as melanoma that were previously considered not only radioresistant but chemoresistant. Table 2 [63-75] summarizes selected clinical trials evaluating the efficacy of targeted therapies alone or in combination with radiation therapy in the treatment of brain metastases. Although, with targeted therapies alone some impressive response rates are observed, progression-free-survival (PFS) rates are usually short (< 8 months). These results are not too dissimilar from what has been achieved with WBRT alone [33, 53]. The exception is in EGFR mutant non-small cell lung cancers treated with a tyrosine kinase inhibitor. In the study by Wu et al. [69] for example, the median PFS was >15 months in EGFR mutant tumours treated with erlotinib.

**Table 2:** Summary of selected trials of targeted therapy alone or in combination with brain radiation for the treatment of brain metastases

Targeted therapy alone								
Trial	Treatment	Disease site	Endpoint	Outcome	Toxicity	PFS	Median OS
Lin et al.^63^ Randomized phase II trial (N=15)	Arm 1: Lapatinib and capecitabine Arm 2: Lapatinub and topotecan	Her2-positive breast cancer with progressive brain metastases after trastuzumab and cranial RT	Objective CNS response (>50% volume reduction)	Response rate: Arm 1= 38% Arm 2= 0%	Closed early due to excess toxicity	NR	NR
Bachelot et al.^64^ LANSCAPE Phase II trial (N=44)	Lapatinib and capecitbine, no previous brain treatment	Her2-positive breast cancer	Proportion of patients with an objective CNS response at 4 weeks (>50% volume reduction)	65.9% (all partial response)	49% had grade 3 or 4	5.5 mos	17 mos
Long et al.^65^ BREAK-MB, Phase II trial (N=172)	Dabrafenib Cohort A: no previous brain treatment Cohort B: progressive disease after brain treatment	Val600Glu or Val600Lys BRAF-mutant melanoma	Proportion of Val600Glu BRAF-mutant patients who achieve overall intracranial response (complete or partial response through modified RECIST)	Cohort A: 39% Cohort B: 31%	22% Grade 3 or 4	Cohort A: 16.1 wks Cohort B: 16.6 wks	Cohort A: 33 wks Cohort B: 31 wks
Margolin et al.^66^ Phase II trial (N=72)	Ipilimumab Cohort A: no steroids and asymptomatic Cohort B: symptomatic on a stable dose of steroid	Metastatic melanoma	Proportion of patients with disease control at 12 weeks (complete response, partial response, or stable disease assessed with modified WHO criteria)	Cohort A: 24% Cohort B: 10%	Cohort A: Grade 3 fatigue (12%) and diarrhea (12%) Cohort B: Grade 3 dehydration (10%) and hyperglycemia (10%)	Cohort A: 1.4 mos Cohort B: 1.2 mos	Cohort A: 7.0 mos Cohort B: 3.7 mos
Gore et al.^67^ Open-label expanded access program (N=213)	Sunitinib	Metastatic RCC	ORR was defined as the number of complete and partial responses according to RECIST	Response rate=12%	Most common grade 3-4 fatigue and asthenia (both 7%)	5.6 mos	9.2 mos
Park et al.^68^ Phase II trial (N=28)	EGFR Tyrosine kinase inhibitor	Exon 19 or 21 mutated EGFR NSCLC	Objective CNS response (complete response, partial response, or stable disease)	83% partial response 11% stable disease	NA	6.6 mos	15.9 mos
Wu et al.^69^ Phase II trial (N=48)	Erlotinib	Asymptomatic brain mets without extracranial progression, after platinum-doublet chemotherapy	PFS determined by RECIST	Median PFS 10.1 mos	Rash 77%, Paronychia 21%,	EGFR wild-type = 4.4 mos EGFR mutant = 15.2 mos	18.9 mos
**Targeted therapy in combination with radiation**								
Staehler et al.^70^ Brain (N=51) Spine (N=55)	Sorafenib or sunitinib and SRS	RCC spine and brain SRS ECOG 0 or 1	Local Control	98% at 15 mos	2 asymptomatic tumour hemorrhage	NA	11.1 mos in brain pts
Lin et al.,^71^ Phase 1 study (N=35)	Lapatinib dose escalating and WBRT (35Gy in 14 fractions)	Her2-positive breast cancer	CNS objective response	Response rate 79%	7/27 pts had DLTs a 1250mg of lapatinib	46% PFS at 6 mos	NR
Welsh et al.^72^ Phase II trial (N=40)	Erlotinib plus WBRT (35Gy in 14 fractions)	NSCLC regardless of EGFR status	CNS objective response according to RECIST	ORR was 86%	Most common Grade 3 toxicity: fatigue (12.5%) and rash (15%)	8.0 mos	Overall 11.8 mos EGFR mutant 19.2 mos
Lee et al.^73^ Phase III trial (N=80)	Arm 1: WBRT and placebo Arm 2: WBRT and erlotinib	NSCLC KPS>70 Multiple brain mets 1/35 EGFR mutant	nPFS	No difference in nPFS	Grade 3 or 4 similar in both arms at 70%	nPFS 1.6 mos in both arms	Arm 1: 2.9 mos Arm 2: 3.4 mos
Sperduto et al.^74^ Phase III trial (N=126)	Arm 1: WBRT and SRS alone Arm 2: WBRT, SRS and TMZ Arm 3: WBRT, SRS and erlotinib	NSCLS with 1-3 brain metastases. No stratification based on predictive biomarkers	Compare OS for TMZ and erlotinib groups versus WBRT and SRS alone group	NA	Grade 3-5 toxicity Arm 1=11%, Arm 2=41%, Arm 3=49% (p<0.001)	Median PFS: Arm1= 8.1 mos Arm 2= 4.6 mos Arm 3= 4.8 mos	Median OS: Arm1: 13.4 mos Arm 2: 6.3 mos Arm 3: 6.1 mos
Knisely et al.^75^ Retrospective review (N=77)	Non-randomized comparison of SRS alone or in combination with ipilimumab	Melanoma brain metastases initially treated with SRS	Compare 2-year and median OS	2-year OS ipilimumab: 47.2% no ipilimumab: 19.7%	NR	NR	Median OS ipilimumab: 21.3mos no ipilimumab: 4.9mos

PFS: progression free survival, nPFS: neurologic progression free survival, OS: overall survival, NSCLC: non-small cell lung cancer, EGFR: epidermal growth factor receptor, WBRT: whole brain radiotherapy, SRS: stereotactic radiosurgery, mos: months, wks: weeks, NS: not significant, NR: not recorded, NA: not applicable

Few trials have combined targeted therapies with radiation, and these are also summarized in Table 2. Although, randomized evidence is still lacking, there is suggestion from retrospective data that the combination may lead to improved outcomes. In the retrospective analysis of a prospective cohort of patients treated with SRS for melanoma brain metastases, the group that received ipilimumab had a significantly longer median survival (21.3 *vs* 4.9 months) [75]. However, caution needs to be exercised as concurrent targeted therapy and radiation treatment may not be as innocuous as previously thought. The RCT evaluating SRS with WBRT alone, versus in combination with erlotinib or temozlomide, reported significantly greater Grade 3 to 5 toxicity rates in the combination arm; more importantly, survival was worse although it did not reach statistical significance [74]. A significant limitation of this study was that the targeted therapy was not biomarker-driven and may in part account for the poor survival observed. Caution also needs to be taken as there is evidence that brain metastases and primary tumors may harbor distinct genetic alterations. In one study of 86 matched brain metastases and primary tumors, clinically informative alterations in 53% of brain metastases were not detected in the primary tumor [76].

The emerging evidence suggests that targeted therapies will play a significant role in the treatment of brain metastases. We postulate that although targeted agents may not be effective in controlling gross disease in the brain, they may be effective in managing micro-metastatic disease in the brain. Therefore, a logical approach may be to combine targeted therapies with SRS alone, overcoming the limitation of SRS in addressing micrometastatic disease in the brain and leading to fewer distant brain failures. The next few years will yield exciting data as there are a number of trials in progress employing this strategy.

## INNOVATIONS IN SRS - INDICATIONS AND APPLICATIONS

**Figure 1:**
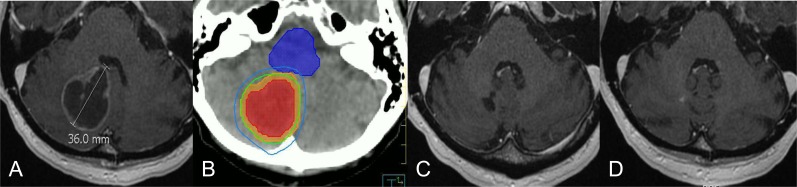
Selected case of large brain metastases treated with hypofractionated stereotactic radiation **A.** MRI of 3.6cm right cerebellar metastases from lung cancer. **B.** Highly conformal treatment plan with the 100% (green line) and 80% (blue line) isodose lines wrapping tightly around the gross tumour (red colorwash), and planning target volume (orange colorwash). The brainstem (blue colorwash) is spared from high dose. (B) MRI 2 months post completion of radiation (30Gy in 5 fractions). **C.** Complete resolution of the tumour at 1 year.

As tumors get larger, SRS dosing is counter-intuitively lowered with respect to tumor control, to maintain safety to the normal brain tissue; otherwise, the risk of radiation necrosis becomes prohibitive [77]. As a result, tumors greater than 4 cm have typically been excluded from single fraction SRS. If the tumors are non-operable, then these patients have been treated with WBRT despite its poor local control. The advent of non-invasive head immobilization devices, on-board image-guidance systems and advanced radiation delivery software has lead to the practice of hypofractionated frameless stereotactic radiotherapy (SRT) [60, 78]. Essentially by fractionating, the safety profile with respect to the normal brain tissue toxicity is improved, and the total tumor dose can be escalated such that doses like 24Gy in 3 fractions and 30-40Gy in 5 fractions are now not unusual. One interesting retrospective series reported higher rates of control and lower rates of toxicities with hypofractionated SRT (36Gy in 6 fractions) as compared to single fraction SRS [79]. However, this practice is still in its infancy and we are in need of a RCT to determine whether or not single fraction SRS should be reserved only for small metastases (e.g. 1-2 centimeters), as the data suggest worse local control for larger tumors [80, 81]. Figure 1 is an example of the efficacy of hypofractionated SRT in a large tumor. This patient would have otherwise been excluded from single fraction SRS and treated with WBRT upfront (plus or minus a SRS boost), or operated upon.

In post-operative patients, the practice of routinely treating with WBRT is also becoming outdated. There is increasing use of SRS to the post-operative surgical cavity as a means to spare patients from the adverse effects of WBRT and to improve local control [82-93]. At the Sunnybrook Odette Cancer Center, we have adopted hypofractionated SRT as our standard of care, delivering 30-35Gy in 5 fractions to the post-operative tumor bed [78]. Fractionation has the advantage of minimizing the adverse effects to the normal tissues, given that the targets tend to be large and irregularly shaped. Our initial results have shown 1 year local control rates of ∼80% in patients who have either recurred and re-operated upon after previous WBRT, or in patients with no prior history of WBRT and operated upon. There is an ongoing study evaluating post-surgical cavity single fraction SRS alone *versus* WBRT and may be a practice-defining study [94].

## CONCLUSIONS

Over the past several decades, clinical trials have informed us about the appropriate management of patients with brain metastases. Initially, SRS was considered an adjunct to WBRT in selected patients presenting with a limited number of brain metastases. However, the role of WBRT has since been questioned as we have learned of its potential to adversely affect QOL and neurocognition. As a result, SRS alone has emerged as the current standard of care with Level 1 evidence to support its practice. Current research is focused on the evaluation of broader applications of SRS to several clinical situations that were traditionally considered absolute indications for WBRT. For example, SRS alone is currently being evaluated in the treatment of 5 or more brain metastases, and in post-operative patients. A new era of trials is also emerging investigating the impact of targeted therapies concurrent with SRS.

Clinical trials in patients with brain metastases are a major challenge as summarized by the recent reports from the Response Assessment in Neuro-Oncology (RANO) brain metastases group [95, 96]. They recommend for trial design and response assessment that trials be tumor or biomarker specific, that assessment be based on contrast-enhanced MRI, that baseline and repeated neurocognitive and QOL testing be performed with validated and sensitive instruments, that stratification be based on extra-cranial disease status, and that appropriate timing be considered with regard to endpoint testing. Ultimately, the oncology community has recognized that the development of brain metastases is no longer the terminal oncologic event of the past.

## References

[1] Tsao MN, Lloyd N, Wong RK, Chow E, Rakovitch E, Laperriere N, Xu W, Sahgal A (2012). Whole brain radiotherapy for the treatment of newly diagnosed multiple brain metastases. Cochrane Database Syst Rev.

[2] Lassman AB, DeAngelis LM (2003). Brain metastases. Neurol Clin.

[3] Barnholtz-Sloan JS, Sloan AE, Davis FG, Vigneau FD, Lai P, Sawaya RE (2004). Incidence proportions of brain metastases in patients diagnosed (1973 to 2001) in the Metropolitan Detroit Cancer Surveillance System. J Clin Oncol.

[4] Patchell RA, Tibbs PA, Walsh JW, Dempsey RJ, Maruyama Y, Kryscio RJ, Markesbery WR, Macdonald JS, Young B (1990). A randomized trial of surgery in the treatment of single metastases to the brain. N Engl J Med.

[5] Vecht CJ, Haaxma-Reiche H, Noordijk EM, Padberg GW, Voormolen JH, Hoekstra FH, Tans JT, Lambooij N, Metsaars JA, Wattendorff AR (1993). Treatment of single brain metastasis: radiotherapy alone or combined with neurosurgery?. Ann Neurol.

[6] Patchell RA, Tibbs PA, Regine WF, Dempsey RJ, Mohiuddin M, Kryscio RJ, Markesbery WR, Foon KA, Young B (1998). Postoperative radiotherapy in the treatment of single metastases to the brain: a randomized trial. JAMA.

[7] Noordijk EM, Vecht CJ, Haaxma-Reiche H, Padberg GW, Voormolen JH, Hoekstra FH, Tans JT, Lambooij N, Metsaars JA, Wattendorff AR (1994). The choice of treatment of single brain metastasis should be based on extracranial tumor activity and age. Int J Radiat Oncol Biol Phys.

[8] Kim MY, Oskarsson T, Acharyya S, Nguyen DX, Zhang XH, Norton L, Massague J (2009). Tumor self-seeding by circulating cancer cells. Cell.

[9] Yu M, Bardia A, Wittner BS, Stott SL, Smas ME, Ting DT, Isakoff SJ, Ciciliano JC, Wells MN, Shah AM, Concannon KF, Donaldson MC, Sequist LV, Brachtel E, Sgroi D, Baselga J (2013). Circulating breast tumor cells exhibit dynamic changes in epithelial and mesenchymal composition. Science.

[10] Pierga JY, Bidard FC, Cropet C, Tresca P, Dalenc F, Romieu G, Campone M, Mahier Ait-Oukhatar C, Le Rhun E, Goncalves A, Leheurteur M, Domont J, Gutierrez M, Cure H, Ferrero JM, Labbe-Devilliers C (2013). Circulating tumor cells and brain metastasis outcome in patients with HER2-positive breast cancer: the LANDSCAPE trial. Ann Oncol.

[11] Ocana OH, Corcoles R, Fabra A, Moreno-Bueno G, Acloque H, Vega S, Barrallo-Gimeno A, Cano A, Nieto MA (2012). Metastatic colonization requires the repression of the epithelial-mesenchymal transition inducer Prrx1. Cancer Cell.

[12] Tsai JH, Donaher JL, Murphy DA, Chau S, Yang J (2012). Spatiotemporal regulation of epithelial-mesenchymal transition is essential for squamous cell carcinoma metastasis. Cancer Cell.

[13] Gunasinghe NP, Wells A, Thompson EW, Hugo HJ (2012). Mesenchymal-epithelial transition (MET) as a mechanism for metastatic colonisation in breast cancer. Cancer Metastasis Rev.

[14] Kienast Y, von Baumgarten L, Fuhrmann M, Klinkert WE, Goldbrunner R, Herms J, Winkler F (2010). Real-time imaging reveals the single steps of brain metastasis formation. Nat Med.

[15] Lorger M, Felding-Habermann B (2010). Capturing changes in the brain microenvironment during initial steps of breast cancer brain metastasis. Am J Pathol.

[16] Carbonell WS, Ansorge O, Sibson N, Muschel R (2009). The vascular basement membrane as “soil” in brain metastasis. PLoS One.

[17] Duda DG, Duyverman AM, Kohno M, Snuderl M, Steller EJ, Fukumura D, Jain RK (2010). Malignant cells facilitate lung metastasis by bringing their own soil. Proc Natl Acad Sci U S A.

[18] Fitzgerald DP, Palmieri D, Hua E, Hargrave E, Herring JM, Qian Y, Vega-Valle E, Weil RJ, Stark AM, Vortmeyer AO, Steeg PS (2008). Reactive glia are recruited by highly proliferative brain metastases of breast cancer and promote tumor cell colonization. Clin Exp Metastasis.

[19] Zhang M, Olsson Y (1995). Reactions of astrocytes and microglial cells around hematogenous metastases of the human brain. Expression of endothelin-like immunoreactivity in reactive astrocytes and activation of microglial cells. J Neurol Sci.

[20] Lin Q, Balasubramanian K, Fan D, Kim SJ, Guo L, Wang H, Bar-Eli M, Aldape KD, Fidler IJ (2010). Reactive astrocytes protect melanoma cells from chemotherapy by sequestering intracellular calcium through gap junction communication channels. Neoplasia.

[21] Kim SJ, Kim JS, Park ES, Lee JS, Lin Q, Langley RR, Maya M, He J, Kim SW, Weihua Z, Balasubramanian K, Fan D, Mills GB, Hung MC, Fidler IJ (2011). Astrocytes upregulate survival genes in tumor cells and induce protection from chemotherapy. Neoplasia.

[22] Seike T, Fujita K, Yamakawa Y, Kido MA, Takiguchi S, Teramoto N, Iguchi H, Noda M (2011). Interaction between lung cancer cells and astrocytes via specific inflammatory cytokines in the microenvironment of brain metastasis. Clin Exp Metastasis.

[23] Denkins Y, Reiland J, Roy M, Sinnappah-Kang ND, Galjour J, Murry BP, Blust J, Aucoin R, Marchetti D (2004). Brain metastases in melanoma: roles of neurotrophins. Neuro Oncol.

[24] Menter DG, Herrmann JL, Nicolson GL (1995). The role of trophic factors and autocrine/paracrine growth factors in brain metastasis. Clin Exp Metastasis.

[25] Rao SS, Thompson C, Cheng J, Haimovitz-Friedman A, Powell SN, Fuks Z, Kolesnick RN (2014). Axitinib sensitization of high Single Dose Radiotherapy. Radiother Oncol.

[26] Finkelstein SE, Timmerman R, McBride WH, Schaue D, Hoffe SE, Mantz CA, Wilson GD (2011). The confluence of stereotactic ablative radiotherapy and tumor immunology. Clin Dev Immunol.

[27] Brown JM, Carlson DJ, Brenner DJ (2014). Dose escalation, not “new biology,” can account for the efficacy of stereotactic body radiation therapy with non-small cell lung cancer. In reply to Rao et al. Int J Radiat Oncol Biol Phys.

[28] Gaspar L, Scott C, Rotman M, Asbell S, Phillips T, Wasserman T, McKenna WG, Byhardt R (1997). Recursive partitioning analysis (RPA) of prognostic factors in three Radiation Therapy Oncology Group (RTOG) brain metastases trials. Int J Radiat Oncol Biol Phys.

[29] Gaspar LE, Scott C, Murray K, Curran W (2000). Validation of the RTOG recursive partitioning analysis (RPA) classification for brain metastases. Int J Radiat Oncol Biol Phys.

[30] Sperduto PW, Kased N, Roberge D, Xu Z, Shanley R, Luo X, Sneed PK, Chao ST, Weil RJ, Suh J, Bhatt A, Jensen AW, Brown PD, Shih HA, Kirkpatrick J, Gaspar LE (2012). Summary report on the graded prognostic assessment: an accurate and facile diagnosis-specific tool to estimate survival for patients with brain metastases. J Clin Oncol.

[31] Kondziolka D, Parry PV, Lunsford LD, Kano H, Flickinger JC, Rakfal S, Arai Y, Loeffler JS, Rush S, Knisely JP, Sheehan J, Friedman W, Tarhini AA, Francis L, Lieberman F, Ahluwalia MS (2014). The accuracy of predicting survival in individual patients with cancer. J Neurosurg.

[32] Diaz LA, Bardelli A (2014). Liquid biopsies: genotyping circulating tumor DNA. J Clin Oncol.

[33] Andrews DW, Scott CB, Sperduto PW, Flanders AE, Gaspar LE, Schell MC, Werner-Wasik M, Demas W, Ryu J, Bahary JP, Souhami L, Rotman M, Mehta MP, Curran WJ (2004). Whole brain radiation therapy with or without stereotactic radiosurgery boost for patients with one to three brain metastases: phase III results of the RTOG 9508 randomised trial. Lancet.

[34] Kondziolka D, Patel A, Lunsford LD, Kassam A, Flickinger JC (1999). Stereotactic radiosurgery plus whole brain radiotherapy versus radiotherapy alone for patients with multiple brain metastases. Int J Radiat Oncol Biol Phys.

[35] Aoyama H, Shirato H, Tago M, Nakagawa K, Toyoda T, Hatano K, Kenjyo M, Oya N, Hirota S, Shioura H, Kunieda E, Inomata T, Hayakawa K, Katoh N, Kobashi G (2006). Stereotactic radiosurgery plus whole-brain radiation therapy vs stereotactic radiosurgery alone for treatment of brain metastases: a randomized controlled trial. JAMA.

[36] Chang EL, Wefel JS, Hess KR, Allen PK, Lang FF, Kornguth DG, Arbuckle RB, Swint JM, Shiu AS, Maor MH, Meyers CA (2009). Neurocognition in patients with brain metastases treated with radiosurgery or radiosurgery plus whole-brain irradiation: a randomised controlled trial. Lancet Oncol.

[37] Kocher M, Soffietti R, Abacioglu U, Villa S, Fauchon F, Baumert BG, Fariselli L, Tzuk-Shina T, Kortmann RD, Carrie C, Ben Hassel M, Kouri M, Valeinis E, van den Berge D, Collette S, Collette L (2011). Adjuvant whole-brain radiotherapy versus observation after radiosurgery or surgical resection of one to three cerebral metastases: results of the EORTC 22952-26001 study. J Clin Oncol.

[38] Brown PD, Asher AL, Ballman KV, Farace E, Cerhan JH, Anderson SK, Carrero XW, Barker FG, Deming RL, Burri S, Menard C, Chung C, Stieber VW, Pollock BE, Galanis E, Buckner JC (2015). NCCTG N0574 (Alliance): A phase III randomized trial of whole brain radiation therapy (WBRT) in addition to radiosurgery (SRS) in patients with 1 to 3 brain metastases. J Clin Oncol (Meeting Abstracts).

[39] Meyers CA, Smith JA, Bezjak A, Mehta MP, Liebmann J, Illidge T, Kunkler I, Caudrelier JM, Eisenberg PD, Meerwaldt J, Siemers R, Carrie C, Gaspar LE, Curran W, Phan SC, Miller RA (2004). Neurocognitive function and progression in patients with brain metastases treated with whole-brain radiation and motexafin gadolinium: results of a randomized phase III trial. J Clin Oncol.

[40] Weiss SE, Kelly PJ (2010). Neurocognitive function after WBRT plus SRS or SRS alone. Lancet Oncol.

[41] Soffietti R, Kocher M, Abacioglu UM, Villa S, Fauchon F, Baumert BG, Fariselli L, Tzuk-Shina T, Kortmann RD, Carrie C, Ben Hassel M, Kouri M, Valeinis E, van den Berge D, Mueller RP, Tridello G (2013). A European Organisation for Research and Treatment of Cancer phase III trial of adjuvant whole-brain radiotherapy versus observation in patients with one to three brain metastases from solid tumors after surgical resection or radiosurgery: quality-of-life results. J Clin Oncol.

[42] Tsao M, Xu W, Sahgal A (2012). A meta-analysis evaluating stereotactic radiosurgery, whole-brain radiotherapy, or both for patients presenting with a limited number of brain metastases. Cancer.

[43] Sahgal A, Aoyama H, Kocher M, Neupane B, Collette S, Tago M, Shaw P, Beyene J, Chang EL (2015). Phase 3 trials of stereotactic radiosurgery with or without whole-brain radiation therapy for 1 to 4 brain metastases: individual patient data meta-analysis. Int J Radiat Oncol Biol Phys.

[44] Auperin A, Arriagada R, Pignon JP, Le Pechoux C, Gregor A, Stephens RJ, Kristjansen PE, Johnson BE, Ueoka H, Wagner H, Aisner J (1999). Prophylactic cranial irradiation for patients with small-cell lung cancer in complete remission. Prophylactic Cranial Irradiation Overview Collaborative Group. N Engl J Med.

[45] Slotman B, Faivre-Finn C, Kramer G, Rankin E, Snee M, Hatton M, Postmus P, Collette L, Musat E, Senan S (2007). Prophylactic cranial irradiation in extensive small-cell lung cancer. N Engl J Med.

[46] Gore EM, Bae K, Wong SJ, Sun A, Bonner JA, Schild SE, Gaspar LE, Bogart JA, Werner-Wasik M, Choy H (2011). Phase III comparison of prophylactic cranial irradiation versus observation in patients with locally advanced non-small-cell lung cancer: primary analysis of radiation therapy oncology group study RTOG 0214. J Clin Oncol.

[47] Sun A, Bae K, Gore EM, Movsas B, Wong SJ, Meyers CA, Bonner JA, Schild SE, Gaspar LE, Bogart JA, Werner-Wasik M, Choy H (2011). Phase III trial of prophylactic cranial irradiation compared with observation in patients with locally advanced non-small-cell lung cancer: neurocognitive and quality-of-life analysis. J Clin Oncol.

[48] Slotman BJ, Mauer ME, Bottomley A, Faivre-Finn C, Kramer GW, Rankin EM, Snee M, Hatton M, Postmus PE, Collette L, Senan S (2009). Prophylactic cranial irradiation in extensive disease small-cell lung cancer: short-term health-related quality of life and patient reported symptoms: results of an international Phase III randomized controlled trial by the EORTC Radiation Oncology and Lung Cancer Groups. J Clin Oncol.

[49] DeAngelis LM, Delattre JY, Posner JB (1989). Radiation-induced dementia in patients cured of brain metastases. Neurology.

[50] Monaco EA, Faraji AH, Berkowitz O, Parry PV, Hadelsberg U, Kano H, Niranjan A, Kondziolka D, Lunsford LD (2013). Leukoencephalopathy after whole-brain radiation therapy plus radiosurgery versus radiosurgery alone for metastatic lung cancer. Cancer.

[51] Lancelot E, Beal MF (1998). Glutamate toxicity in chronic neurodegenerative disease. Prog Brain Res.

[52] Brown PD, Pugh S, Laack NN, Wefel JS, Khuntia D, Meyers C, Choucair A, Fox S, Suh JH, Roberge D, Kavadi V, Bentzen SM, Mehta MP, Watkins-Bruner D (2013). Memantine for the prevention of cognitive dysfunction in patients receiving whole-brain radiotherapy: a randomized, double-blind, placebo-controlled trial. Neuro Oncol.

[53] Gondi V, Pugh SL, Tome WA, Caine C, Corn B, Kanner A, Rowley H, Kundapur V, DeNittis A, Greenspoon JN, Konski AA, Bauman GS, Shah S, Shi W, Wendland M, Kachnic L (2014). Preservation of memory with conformal avoidance of the hippocampal neural stem-cell compartment during whole-brain radiotherapy for brain metastases (RTOG 0933): a phase II multi-institutional trial. J Clin Oncol.

[54] Tsao MN, Rades D, Wirth A, Lo SS, Danielson BL, Gaspar LE, Sperduto PW, Vogelbaum MA, Radawski JD, Wang JZ, Gillin MT, Mohideen N, Hahn CA, Chang EL (2012). Radiotherapeutic and surgical management for newly diagnosed brain metastasis(es): An American Society for Radiation Oncology evidence-based guideline. Pract Radiat Oncol.

[55] ChoosingWisely (2014). ASTRO releases second list of five radiation oncology treatments to question, as part of national Choosing Wisely campaign. http://www.choosingwisely.org/astro-releases-second-list/.

[56] Nabors LB, Portnow J, Ammirati M, Brem H, Brown P, Butowski N, Chamberlain MC, DeAngelis LM, Fenstermaker RA, Friedman A, Gilbert MR, Hattangadi-Gluth J, Hesser D, Holdhoff M, Junck L, Lawson R (2014). Central nervous system cancers, version 2.2014. Featured updates to the NCCN Guidelines. J Natl Compr Canc Netw.

[57] Bhatnagar AK, Flickinger JC, Kondziolka D, Lunsford LD (2006). Stereotactic radiosurgery for four or more intracranial metastases. Int J Radiat Oncol Biol Phys.

[58] Baschnagel AM, Meyer KD, Chen PY, Krauss DJ, Olson RE, Pieper DR, Maitz AH, Ye H, Grills IS (2013). Tumor volume as a predictor of survival and local control in patients with brain metastases treated with Gamma Knife surgery. J Neurosurg.

[59] Kondziolka D, Kano H, Harrison GL, Yang HC, Liew DN, Niranjan A, Brufsky AM, Flickinger JC, Lunsford LD (2011). Stereotactic radiosurgery as primary and salvage treatment for brain metastases from breast cancer. Clinical article. J Neurosurg.

[60] Sahgal A, Ma L, Chang E, Shiu A, Larson DA, Laperriere N, Yin FF, Tsao M, Menard C, Basran P, Letourneau D, Heydarian M, Beachey D, Shukla V, Cusimano M, Hodaie M (2009). Advances in technology for intracranial stereotactic radiosurgery. Technol Cancer Res Treat.

[61] Thomas EM, Popple RA, Wu X, Clark GM, Markert JM, Guthrie BL, Yuan Y, Dobelbower MC, Spencer SA, Fiveash JB (2014). Comparison of plan quality and delivery time between volumetric arc therapy (RapidArc) and Gamma Knife radiosurgery for multiple cranial metastases. Neurosurgery.

[62] Yamamoto M, Serizawa T, Shuto T, Akabane A, Higuchi Y, Kawagishi J, Yamanaka K, Sato Y, Jokura H, Yomo S, Nagano O, Kenai H, Moriki A, Suzuki S, Kida Y, Iwai Y (2014). Stereotactic radiosurgery for patients with multiple brain metastases (JLGK0901): a multi-institutional prospective observational study. Lancet Oncol.

[63] Lin NU, Eierman W, Greil R, Campone M, Kaufman B, Steplewski K, Lane SR, Zembryki D, Rubin SD, Winer EP (2011). Randomized phase II study of lapatinib plus capecitabine or lapatinib plus topotecan for patients with HER2-positive breast cancer brain metastases. J Neurooncol.

[64] Bachelot T, Romieu G, Campone M, Dieras V, Cropet C, Dalenc F, Jimenez M, Le Rhun E, Pierga JY, Goncalves A, Leheurteur M, Domont J, Gutierrez M, Cure H, Ferrero JM, Labbe-Devilliers C (2013). Lapatinib plus capecitabine in patients with previously untreated brain metastases from HER2-positive metastatic breast cancer (LANDSCAPE): a single-group phase 2 study. Lancet Oncol.

[65] Long GV, Trefzer U, Davies MA, Kefford RF, Ascierto PA, Chapman PB, Puzanov I, Hauschild A, Robert C, Algazi A, Mortier L, Tawbi H, Wilhelm T, Zimmer L, Switzky J, Swann S (2012). Dabrafenib in patients with Val600Glu or Val600Lys BRAF-mutant melanoma metastatic to the brain (BREAK-MB): a multicentre, open-label, phase 2 trial. Lancet Oncol.

[66] Margolin K, Ernstoff MS, Hamid O, Lawrence D, McDermott D, Puzanov I, Wolchok JD, Clark JI, Sznol M, Logan TF, Richards J, Michener T, Balogh A, Heller KN, Hodi FS (2012). Ipilimumab in patients with melanoma and brain metastases: an open-label, phase 2 trial. Lancet Oncol.

[67] Gore ME, Hariharan S, Porta C, Bracarda S, Hawkins R, Bjarnason GA, Oudard S, Lee SH, Carteni G, Nieto A, Yuan J, Szczylik C (2011). Sunitinib in metastatic renal cell carcinoma patients with brain metastases. Cancer.

[68] Park SJ, Kim HT, Lee DH, Kim KP, Kim SW, Suh C, Lee JS (2012). Efficacy of epidermal growth factor receptor tyrosine kinase inhibitors for brain metastasis in non-small cell lung cancer patients harboring either exon 19 or 21 mutation. Lung Cancer.

[69] Wu YL, Zhou C, Cheng Y, Lu S, Chen GY, Huang C, Huang YS, Yan HH, Ren S, Liu Y, Yang JJ (2013). Erlotinib as second-line treatment in patients with advanced non-small-cell lung cancer and asymptomatic brain metastases: a phase II study (CTONG-0803). Ann Oncol.

[70] Staehler M, Haseke N, Nuhn P, Tullmann C, Karl A, Siebels M, Stief CG, Wowra B, Muacevic A (2011). Simultaneous anti-angiogenic therapy and single-fraction radiosurgery in clinically relevant metastases from renal cell carcinoma. BJU Int.

[71] Lin NU, Freedman RA, Ramakrishna N, Younger J, Storniolo AM, Bellon JR, Come SE, Gelman RS, Harris GJ, Henderson MA, Macdonald SM, Mahadevan A, Eisenberg E, Ligibel JA, Mayer EL, Moy B (2013). A phase I study of lapatinib with whole brain radiotherapy in patients with Human Epidermal Growth Factor Receptor 2 (HER2)-positive breast cancer brain metastases. Breast Cancer Res Treat.

[72] Welsh JW, Komaki R, Amini A, Munsell MF, Unger W, Allen PK, Chang JY, Wefel JS, McGovern SL, Garland LL, Chen SS, Holt J, Liao Z, Brown P, Sulman E, Heymach JV (2013). Phase II trial of erlotinib plus concurrent whole-brain radiation therapy for patients with brain metastases from non-small-cell lung cancer. J Clin Oncol.

[73] Lee SM, Lewanski CR, Counsell N, Ottensmeier C, Bates A, Patel N, Wadsworth C, Ngai Y, Hackshaw A, Faivre-Finn C (2014). Randomized trial of erlotinib plus whole-brain radiotherapy for NSCLC patients with multiple brain metastases. J Natl Cancer Inst.

[74] Sperduto PW, Wang M, Robins HI, Schell MC, Werner-Wasik M, Komaki R, Souhami L, Buyyounouski MK, Khuntia D, Demas W, Shah SA, Nedzi LA, Perry G, Suh JH, Mehta MP (2013). A phase 3 trial of whole brain radiation therapy and stereotactic radiosurgery alone versus WBRT and SRS with temozolomide or erlotinib for non-small cell lung cancer and 1 to 3 brain metastases: Radiation Therapy Oncology Group 0320. Int J Radiat Oncol Biol Phys.

[75] Knisely JP, Yu JB, Flanigan J, Sznol M, Kluger HM, Chiang VL (2012). Radiosurgery for melanoma brain metastases in the ipilimumab era and the possibility of longer survival. J Neurosurg.

[76] Brastianos PK, Carter SL, Santagata S, Cahill DP, Taylor-Weiner A, Jones RT, Van Allen EM, Lawrence MS, Horowitz PM, Cibulskis K, Ligon KL, Tabernero J, Seoane J, Martinez-Saez E, Curry WT, Dunn IF (2015). Genomic Characterization of Brain Metastases Reveals Branched Evolution and Potential Therapeutic Targets. Cancer Discov.

[77] Shaw E, Scott C, Souhami L, Dinapoli R, Kline R, Loeffler J, Farnan N (2000). Single dose radiosurgical treatment of recurrent previously irradiated primary brain tumors and brain metastases: final report of RTOG protocol 90-05. Int J Radiat Oncol Biol Phys.

[78] Al-Omair A, Soliman H, Xu W, Karotki A, Mainprize T, Phan N, Das S, Keith J, Yeung R, Perry J, Tsao M, Sahgal A (2013). Hypofractionated stereotactic radiotherapy in five daily fractions for post-operative surgical cavities in brain metastases patients with and without prior whole brain radiation. Technol Cancer Res Treat.

[79] Kim YJ, Cho KH, Kim JY, Lim YK, Min HS, Lee SH, Kim HJ, Gwak HS, Yoo H (2011). Single-dose versus fractionated stereotactic radiotherapy for brain metastases. Int J Radiat Oncol Biol Phys.

[80] Chang EL, Hassenbusch SJ, Shiu AS, Lang FF, Allen PK, Sawaya R, Maor MH (2003). The role of tumor size in the radiosurgical management of patients with ambiguous brain metastases. Neurosurgery.

[81] Follwell MJ, Khu KJ, Cheng L, Xu W, Mikulis DJ, Millar BA, Tsao MN, Laperriere NJ, Bernstein M, Sahgal A (2012). Volume specific response criteria for brain metastases following salvage stereotactic radiosurgery and associated predictors of response. Acta Oncol.

[82] Roberge D, Parney I, Brown PD (2012). Radiosurgery to the postoperative surgical cavity: who needs evidence?. Int J Radiat Oncol Biol Phys.

[83] Iwai Y, Yamanaka K, Yasui T (2008). Boost radiosurgery for treatment of brain metastases after surgical resections. Surg Neurol.

[84] Do L, Pezner R, Radany E, Liu A, Staud C, Badie B (2009). Resection followed by stereotactic radiosurgery to resection cavity for intracranial metastases. Int J Radiat Oncol Biol Phys.

[85] Mathieu D, Kondziolka D, Flickinger JC, Fortin D, Kenny B, Michaud K, Mongia S, Niranjan A, Lunsford LD (2008). Tumor bed radiosurgery after resection of cerebral metastases. Neurosurgery.

[86] Karlovits BJ, Quigley MR, Karlovits SM, Miller L, Johnson M, Gayou O, Fuhrer R (2009). Stereotactic radiosurgery boost to the resection bed for oligometastatic brain disease: challenging the tradition of adjuvant whole-brain radiotherapy. Neurosurg Focus.

[87] Jagannathan J, Yen CP, Ray DK, Schlesinger D, Oskouian RJ, Pouratian N, Shaffrey ME, Larner J, Sheehan JP (2009). Gamma Knife radiosurgery to the surgical cavity following resection of brain metastases. J Neurosurg.

[88] Limbrick DD, Lusis EA, Chicoine MR, Rich KM, Dacey RG, Dowling JL, Grubb RL, Filiput EA, Drzymala RE, Mansur DB, Simpson JR (2009). Combined surgical resection and stereotactic radiosurgery for treatment of cerebral metastases. Surg Neurol.

[89] Brennan C, Yang TJ, Hilden P, Zhang Z, Chan K, Yamada Y, Chan TA, Lymberis SC, Narayana A, Tabar V, Gutin PH, Ballangrud A, Lis E, Beal K (2014). A phase 2 trial of stereotactic radiosurgery boost after surgical resection for brain metastases. Int J Radiat Oncol Biol Phys.

[90] Hartford AC, Paravati AJ, Spire WJ, Li Z, Jarvis LA, Fadul CE, Rhodes CH, Erkmen K, Friedman J, Gladstone DJ, Hug EB, Roberts DW, Simmons NE (2013). Postoperative stereotactic radiosurgery without whole-brain radiation therapy for brain metastases: potential role of preoperative tumor size. Int J Radiat Oncol Biol Phys.

[91] Kalani MY, Filippidis AS, Kalani MA, Sanai N, Brachman D, McBride HL, Shetter AG, Smith KA (2010). Gamma Knife surgery combined with resection for treatment of a single brain metastasis: preliminary results. J Neurosurg.

[92] Choi CY, Chang SD, Gibbs IC, Adler JR, Harsh GRt, Lieberson RE, Soltys SG (2012). Stereotactic radiosurgery of the postoperative resection cavity for brain metastases: prospective evaluation of target margin on tumor control. Int J Radiat Oncol Biol Phys.

[93] Soltys SG, Adler JR, Lipani JD, Jackson PS, Choi CY, Puataweepong P, White S, Gibbs IC, Chang SD (2008). Stereotactic radiosurgery of the postoperative resection cavity for brain metastases. Int J Radiat Oncol Biol Phys.

[94] Clinicaltrials.gov Stereotactic Radiosurgery or Whole-Brain Radiation Therapy in Treating Patients With Brain Metastases That Have Been Removed By Surgery. https://clinicaltrials.gov/show/NCT01372774.

[95] Lin NU, Lee EQ, Aoyama H, Barani IJ, Baumert BG, Brown PD, Camidge DR, Chang SM, Dancey J, Gaspar LE, Harris GJ, Hodi FS, Kalkanis SN, Lamborn KR, Linskey ME, Macdonald DR (2013). Challenges relating to solid tumour brain metastases in clinical trials, part 1: patient population, response, and progression. A report from the RANO group. Lancet Oncol.

[96] Lin NU, Wefel JS, Lee EQ, Schiff D, van den Bent MJ, Soffietti R, Suh JH, Vogelbaum MA, Mehta MP, Dancey J, Linskey ME, Camidge DR, Aoyama H, Brown PD, Chang SM, Kalkanis SN (2013). Challenges relating to solid tumour brain metastases in clinical trials, part 2: neurocognitive, neurological, and quality-of-life outcomes. A report from the RANO group. Lancet Oncol.

